# Case series of Oropouche fever among travellers returning from Cuba to Spain, 2024

**DOI:** 10.2807/1560-7917.ES.2025.30.41.2400719

**Published:** 2025-10-16

**Authors:** Nuria Labiod, Mª Paz Sánchez-Seco, Josune Goikoetxea, Nelly Daniela Zurita, Francesca F Norman, Miguel M Martínez, Pedro Alonso Alonso, Araceli Hernández-Betancor, Marco Antonio Sempere-Alcocer, Zaira Moure, Maria Dolores Ocete, Maria Rosario Vicente, Fernando de la Calle-Prieto, Mikel Gallego, Laura Cardeñoso Domingo, Juan Carlos Galán, Daniel Camprubí-Ferrer, Carlos Meilán, Isabel Perez-Hernandez, Itxasne Cabezón, Francisco Javier Hernández, Enrique Bernal, Ana Vázquez, on behalf of the Spanish-OROV Study Group

**Affiliations:** 1 Arboviruses and Viral Imported Diseases Laboratory, Centro Nacional de Microbiología, Instituto de Salud Carlos III, Majadahonda, Spain; 2 Centro de Investigación Biomédica en Red de Enfermedades Infecciosas (CIBERINFEC), Madrid, Spain; 3 Infectious Diseases Department, Hospital Universitario de Cruces, Instituto de investigación sanitaria Biobizkaia, Spain; 4 Servicio de Microbiología del Hospital Universitario de La Princesa, Madrid, Spain; 5 National Referral Unit for Tropical Diseases, Infectious Diseases Department, Ramón y Cajal University Hospital, Madrid, Spain; 6 Instituto Ramón y Cajal de Investigación Sanitaria (IRYCIS), Madrid, Spain; 7 Universidad de Alcalá, Madrid, Spain; 8 Microbiology Department, Hospital Clínic-Universitat de Barcelona, Barcelona, Spain; 9 Barcelona Institute for Global Health (ISGlobal), Hospital Clínic-Universitat de Barcelona, Barcelona, Spain; 10 Servicio Microbiología, Hospital Monforte de Lemos, Lugo, Spain; 11 Servicio de Microbiología, Hospital Universitario Insular de Gran Canaria (HUIGC), Las Palmas, Spain; 12 Clinical Microbiology Department. Hospital Universitario Virgen de la Victoria, Málaga, Spain; 13 Universidad Internacional de la Rioja, Health Sciences School, Logroño, Spain; 14 Clinical Microbiology Department, Hospital Universitario Marqués de Valdecilla, Instituto de investigación sanitaria Valdecilla (IDIVAL), Santander, Spain; 15 Microbiology Department, Hospital General Universitario de Valencia, Valencia, Spain; 16 Sección de Enfermedades Infecciosas. Hospital General Universitario Reina Sofia. Murcia, Spain; 17 Centro de Referencia Nacional para Patología Importada y Salud Internacional, Adultos y Pediatría, Hospital La Paz-Carlos III, Madrid, Spain; 18 Instituto de Investigación Hospital Universitario La Paz (IdiPAZ), Madrid, Spain; 19 Clinical Microbiology Department, Hospital Universitario de Cruces, Instituto de Investigación Sanitaria Biobizkaia, Barakaldo, Spain; 20 Centro de Investigación Biomédica en Red de Epidemiología y Salud Pública (CIBERESP), Madrid, Spain; 21 Servicio de Microbiología Hospital Ramón y Cajal, Madrid, Spain; 22 International Health Department, Hospital Clínic de Barcelona, Barcelona, Spain; 23 Facultat de Medicina i Ciències de la Salut, Universitat de Barcelona (UB), Barcelona, Spain; 24 Emergency Department, Hospital Monforte de Lemos, Lugo, Spain; 25 Unidad de Enfermedades Infecciosas. Hospital Universitario Virgen de la Victoria, Málaga, Spain; 26 IBIMA-Plataforma BIONAND, Málaga, Spain; 27 Infectious Diseases Department, Hospital Universitario Marqués de Valdecilla, Instituto de Investigación Sanitaria Valdecilla (IDIVAL), Santander, Spain; 28 Instituto Murciano de Investigación Biosanitaria (IMIB), Universidad de Murcia, Murcia, Spain; 29 The members of the Spanish OROV Study Group are listed under Acknowledgements

**Keywords:** Oropouche virus, arbovirus, diagnosis, Imported cases, Emergence, Epidemiological Surveillance

## Abstract

Oropouche fever is a vector-borne disease endemic in Central and South America. Infection with Oropouche virus (OROV) was confirmed in June and July 2024 in 13 patients travelling from Cuba to Spain. These patients presented fever, headache, myalgia and arthralgia, and three patients exhibited a biphasic course, with two developing transient neurological symptoms. Oropouche virus infection was diagnosed using reverse transcription quantitative PCR (RT-qPCR) and neutralisation tests. Viral RNA was detected in specimens from serum, urine, plasma and whole blood; from four urine samples up to 24 days post-symptom onset. Phylogenetic analysis of one OROV strain (OROV SP2024) isolated from one patient, demonstrated it clustered closely with reassortant strains circulating in Brazil and imported cases in Italy. These findings underscore the importance of including OROV in the differential diagnosis of febrile illnesses in returning travellers and demonstrate the diagnostic value of analysing multiple sample types. Enhanced clinical awareness and diagnostic capacity are essential to improve detection and surveillance of OROV among international travellers from affected regions.

**Key public health message**

**What did you want to address in this study and why?**

Oropouche fever, a febrile illness, is caused by Oropouche virus (OROV) and transmitted by midges and mosquitoes. The disease occurs in Central and South America. In June and July 2024, OROV infection was detected in travellers returning from Cuba to Spain. Here we describe the detection of OROV in febrile patients and clinical course of the disease in these patients.

**What have we learned from this study?**

Thirteen patients with OROV infection, recently travelled from Cuba to Spain, presented with fever, headache and joint pain, two of them had temporary neurological symptoms. Testing various sample types (serum, urine, plasma and whole blood) was useful for diagnosis of the infection. The viral RNA was detectable for up to 24 days in a urine sample.

**What are the implications of its findings for public health?**

Healthcare professionals should consider OROV infection when diagnosing febrile patients returning from countries where OROV is circulating. Strengthening collaboration and information sharing between clinicians and public health laboratories is important to improve surveillance and diagnosis of OROV. In suspected cases, analysing different sample types may be beneficial, particularly when a longer time has elapsed since symptom onset.

## Background

Oropouche virus (OROV) was isolated for the first time in Trinidad and Tobago in 1955 [1] and has since then been detected in Bolivia, Brazil [2], Colombia [3], Cuba [4], Ecuador [5], French Guiana (France) [6], Panama [7] and Peru [8]. Endemic in Central and South America, OROV is a zoonotic virus belonging to the genus of *Orthobunyavirus,* family *Peribunyaviridae* [9] and classified into four genotypes (I, II, III and IV) [10].

Despite its circulation mostly confined to the areas previously mentioned, an outbreak occurred in Brazil between 2023 and 2024, including a considerable expansion into regions where the virus had not previously been detected [11]. During 2024, 16,239 confirmed cases were reported in the following countries: Barbados (n = 2), Bolivia (n = 356), Brazil (n = 13,785), Colombia (n = 74), Cuba (n = 626), Ecuador (n = 3), Guyana (n = 3), Panama (n = 16), Peru (n = 1,263), and, for the first time, imported cases were reported in Europe (Germany: n = 3, Italy: n = 6, Spain: n = 21), Canada (n = 2), the United States (US) (n = 108) and the Cayman Islands, a British Overseas Territory (n = 1) [12-15].

Transmission of OROV occurs in both urban and sylvatic cycles, with *Culicoides paraensis* midge serving as the primary vector [16-19]; however, *Culex quinquefasciatus* mosquito has also been implicated in urban transmission cycles in Brazil and French Guiana, with recent detection of OROV RNA in *Cx. quinquefasciatus* in 2024 in Cuba [4,20,21].

The clinical picture of OROV infection resembles that of infections with other arboviruses such as dengue (DENV), Zika (ZIKV) and chikungunya (CHIKV) virus, complicating the clinical diagnosis [22]. The clinical course typically begins with an acute febrile syndrome and nonspecific symptoms, followed by spontaneous recovery within approximately 7 days [20]. However, biphasic courses have been described in which patients experience a relapse of symptoms after apparent remission, sometimes accompanied by mild to moderate neurological manifestations such as meningitis or meningoencephalitis [21,23].

For the molecular diagnosis of OROV, reverse transcription PCR (RT-PCR) and real-time quantitative (RT-qPCR) are used [24-27]. For serological diagnosis, detection of IgM and IgG antibodies by ELISA and immunofluorescence assays are generally used and the neutralisation test to detect neutralising antibodies [10,28].

## Outbreak detection

On 9 May 2024, Pan American Health Organisation (PAHO) issued an epidemiological alert for Oropouche fever in the Region of the Americas, prompted by the reports of > 13,000 confirmed cases of OROV disease, including two deaths, reports of OROV disease cases outside Brazil’s Amazon region and the high co-circulation of DENV in several countries and territories. In response, PAHO, in collaboration with World Health Organization (WHO), recommended WHO member states to immediately implement laboratory methods for the differential diagnosis of OROV [29,30].

Following the PAHO alert, the National Reference Laboratory (NRL) for Zoonosis in Spain, the National Center for Microbiology (NCM), set up specific OROV diagnostic techniques, including RT-qPCR and neutralisation assays. Several Spanish hospitals started sending samples from febrile travellers with suspected OROV infections to the NRL for molecular and serological diagnosis.

## Methods

We collected detailed demographic, clinical and travel history data from travellers from Latin American countries prospectively and retrospectively. Data were obtained from laboratory reports and hospital records, and these included information about age, sex, travel itineraries and symptom onset.

### Case definitions

We received samples from travellers returning to Spain from areas with reported active circulation of OROV. The case definitions are described in Box.

Box **Case definitions of Oropouche virus infection among travellers returning to Spain, 2024**

**Suspected case:**

• Sudden onset of fever (≥ 38°C) and symptoms compatible with an arbovirus infection, such as headache, myalgia, nausea and arthralgia

OR

• Atypical symptoms or complications, including neurological manifestations

AND

• History of travel to areas with OROV circulation.

**Confirmed case:**

• Suspected case

AND

• Detection of OROV by RT-qPCR or serological response confirmed by neutralisation assay.

OROV: Oropouche virus; RT-qPCR: reverse transcription real-time quantitative PCR.

### Data collection

#### Prospective study

We analysed blood, serum, plasma, cerebrospinal fluid (CSF) and urine samples by RT-qPCR from travellers returning from South and Central American countries between 1 June and 31 July 2024. If a strong clinical and epidemiological suspicion was raised but the PCR results were negative, convalescent-phase serum samples for serological testing were collected 1 month after symptom onset.

#### Retrospective study

Additionally, acute samples from returning travellers previously tested negative for DENV, ZIKV and CHIKV between 1 February and 31 May 2024 were analysed for OROV by RT-qPCR. No serological test was performed because all analysed samples were collected during the acute phase of the illness.

### Microbiological investigations

#### Molecular testing

We used a multiplex RT-qPCR assay to simultaneously detect OROV and Mayaro virus (MAYV) [26]. All samples with a quantification cycle (Cq) value < 38 were considered positive and were confirmed by analysing other samples from the same patient or by a second analysis of the same sample. For other arboviral infections, RT-qPCR was used to detect DENV, ZIKV and CHIKV.

#### Virus isolation

The positive samples were inoculated into Vero E6 cells (CRL 1586, American Type Culture Collection (ATCC), https://www.atcc.org), incubated at 37°C for 5 days after which cytopathic effects were observed using a microscope. For virus titration, the mean tissue culture infectious dose (TCID50) was used, i.e. the dilution of a virus required to infect 50% of a given cell culture [31].

#### Serological assay

In the absence of commercially available specific serological methods for diagnosis of OROV infection, a neutralisation assay (NT) was implemented and used to confirm infections when RT-qPCR results were negative or to corroborate the immune response during the convalescence phase. To determine the neutralising activity of the sera, NT was carried out using a viral isolate obtained in this study from a serum sample of one patient (OROV SP2024). Serum samples were diluted from 1:8 to 1:512 and incubated with the virus at 37°C and 5% CO_2_ for 1 h. Then, Vero cells were seeded onto 96 plates at a concentration of 4 × 10^5^ cells/mL, containing the virus-serum mixture and incubated in the same conditions. The plates were evaluated for cytopathic effect with a microscope after an incubation of 3 and 4 days. The titres of neutralising antibodies were defined as the highest serum dilution that showed > 90% neutralisation of the virus [32]. The method was validated using a convalescent serum from a patient with a previously positive RT-qPCR result in acute samples.

#### Sequencing and phylogenetic analysis

We used next-generation sequencing (NGS) techniques to obtain the complete genome of the isolate OROV SP2024 and compared its phylogenetic relationship with strains from recent outbreaks in Brazil and imported cases in other countries. The libraries were prepared using the NEBNext Ultra II RNA Library Prep-Kit (New England Biolabs, Ipswich, US) with NEBNext Multiplex Oligos (New England Biolabs) and sequenced directly on an Illumina MiSeq Reagent Nano kit v2 (300 cycles) (Illumina, San Diego, US). Sequencing data were analysed for viral genome reconstruction using viralrecon pipeline version 2.6.0 (https://github.com/nf-core/viralrecon). Detection of variants was performed using ivar variants version 1.4 (https://github.com/andersen-lab/ivar) and viral genome consensus was generated using BEDtools version 2.30.0 (https://bedtools.readthedocs.io) and variants with an allele frequency > 80% and masking genomic regions with coverage values < 10 reads. The complete nucleotide sequences are available in GenBank (https://www.ncbi.nlm.nih.gov/genbank). The evolutionary history was inferred using the Neighbour-Joining method and Tamura-Nei model for the distances in Molecular Evolutionary genetic Analysis (MEGA) version 11 (https://www.megasoftware.net). Bootstrap analyses were performed using 1,000 bootstrap replications.

## Results

Of the 144 travellers tested, 13 (9.0%) were diagnosed with OROV infection. In the prospective sampling, 39 samples were analysed from 27 travellers coming from Cuba (n = 21), Peru (n = 1) and other Central and South American countries (n = 5), and OROV was detected in 12 patients. In the retrospective sampling, OROV was detected in one (0.9%) of 117 travellers arriving from Argentina, Bolivia, Brazil, Colombia, Cuba, Peru and Venezuela. All confirmed cases had travelled to Cuba between 1 May and 31 July 2024, coinciding with the active circulation of the virus in this region. Nine cases were males and four were females, the ages ranging from their twenties to their sixties.

**Figure 1:**
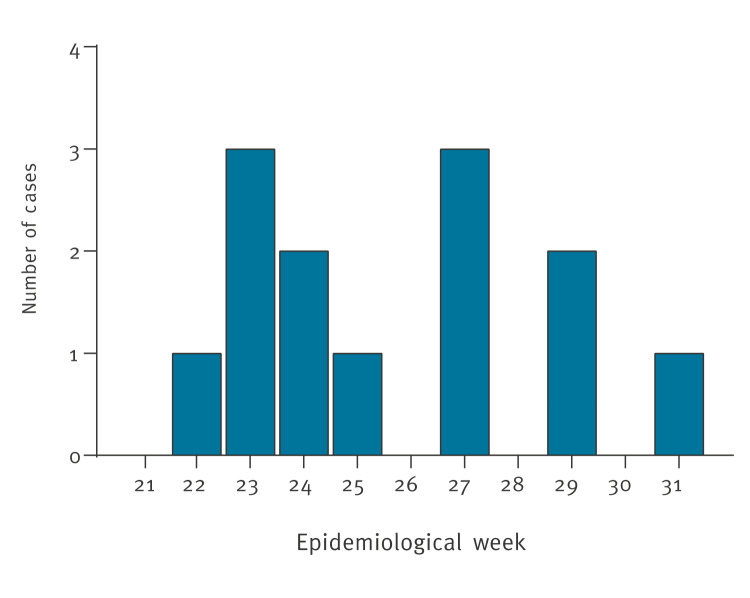
Detection of Oropouche virus infection in travellers returning from Cuba, by week, Spain, May–July 2024 (n = 13)^a^

^a^ Number of confirmed imported OROV cases by epidemiological week (weeks 21–31).

The symptoms of all cases started either during their stay in Cuba or within a few days after returning to Spain. The first case was diagnosed on 2 June 2024, the others later in June and July (epidemiological weeks 22–31) (Figure 1). The confirmed cases were detected in 9 of the 17 autonomous communities of Spain.

### Clinical picture

**Figure 2:**
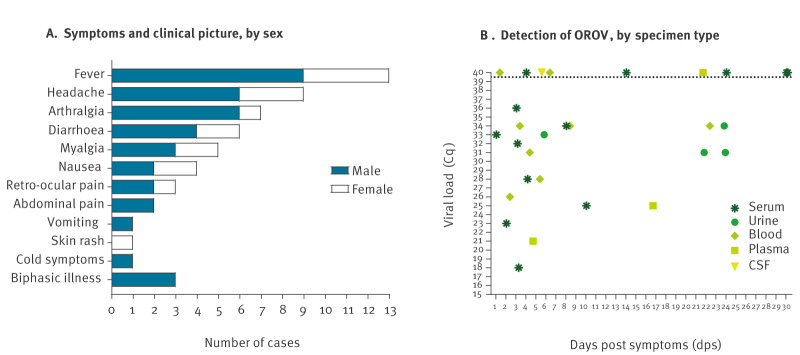
Clinical picture of Oropouche virus infection, by sex (A) and detection of Oropouche virus, by specimen type (B) in travellers returning from Cuba, Spain, 2024 (n = 13)

All confirmed cases had fever (Figure 2A). The other symptoms were as follows: headache (n = 9), arthralgia (n = 7), diarrhoea (n = 4), myalgia (n = 5), retro-ocular pain (n = 3), abdominal pain (n = 2), neurological symptoms (n = 2) and vomiting, skin rash and cold symptoms (n = 1).

Cq: quantification cycle; CSF: cerebrospinal fluid; OROV: Oropouche virus; RT-qPCR: reverse transcription real-time quantitative PCR.

Panel A. More than one symptom could be reported per patient. Nine males and four females were included in the analysis.

Panel B. More than one specimen type could be analysed per patient. RT-qPCR Cq values are plotted against days post symptom onset (dps). Cq values < 38 were considered positive.

Three confirmed cases (Case A, B and C) had a biphasic course of the disease. Cases A and B did not have underlying conditions, Case C had hypertension and diabetes.

First, Case A, a male in his thirties, presented with fever, headache, retro-ocular pain, myalgia, arthralgia and diarrhoea, then reported resolution of symptoms, but after 2 days the symptoms recurred, marked by the onset of a skin rash. Case B and C had neurological complications. For Case B, a male in his twenties, the first symptoms were a febrile illness with temperatures exceeding 39°C, general malaise, arthralgia/myalgia and increased bowel movements. The symptoms resolved, but after 15 afebrile days, the symptoms recurred in addition to intense headache, vertigo, postural and gait instability and a subjective sensation of diplopia that required 24 h of observation. Case C, a male in his fifties, 4 days after arriving to Cuba, presented with high fever, arthralgia, myalgia, malaise, headache and diarrhoea. The symptoms resolved spontaneously after 1 week, but 11 days later, all symptoms except for diarrhoea reappeared and neurological symptoms of tactile allodynia and hyperalgesia on the left side of the body appeared.

Other clinical complications were also observed. A female in her fifties, Case D, presented with persistent myalgia and headache for up to 20 days, without severe clinical signs or long-term sequelae, and another patient, a male in his sixties, Case E, presented with mild hepatic involvement (elevation of liver enzymes), accompanied by nonspecific upper respiratory symptoms.

Case F, a male in his forties, identified in the retrospective sampling, developed cold- and dengue-like symptoms without a rash, including diarrhoea, nausea and ocular pain, followed by abdominal pain and fever. Based on clinical suspicion, dengue was initially considered; however, diagnostic tests for DENV, CHIKV and ZIKV were negative. The case was subsequently confirmed as an OROV infection by RT-qPCR.

One confirmed case was hospitalised and recovered spontaneously without complications.

### Laboratory analyses

Twelve cases tested positive for OROV by qRT-PCR and one case was diagnosed serologically. Viral RNA was detected in multiple sample types on various days post-symptom onset (dps) (Figure 2B). Serum was the most common specimen type. Serum samples from 8 of 12 confirmed cases were positive for OROV, mainly during the first 10 days, with Cq values generally < 33. Oropouche virus was also detected from urine (4/4) and plasma (2/3) samples of confirmed cases. In plasma samples, viral RNA was detectable for up to 17 dps and in urine samples for up to 24 dps, i.e. longer than in serum samples. The urine samples were from Cases A–C and from a patient with persistent symptoms. One urine sample was positive for OROV 24 days after the onset of symptoms. Blood samples were also tested (7/8), showing similar detection patterns to serum. A CSF sample collected during the early phase of the disease (≤ 6 dps) was also analysed and was negative with a Ct value > 39.

Two individuals who travelled together to Cuba with a shared epidemiological link developed symptoms 1 week apart from each other. Oropouche virus was detected with RT-qPCR in samples of one of these two cases whereas the samples of the other case tested negative. Due to the strong clinical suspicion and the epidemiological link, paired acute and convalescent serum samples from both patients were tested by neutralisation assay. Both individuals had neutralising antibody titres of 1:64 in convalescent samples, while acute phase samples were negative. This case was the only prospectively confirmed case based on serology.

**Figure 3:**
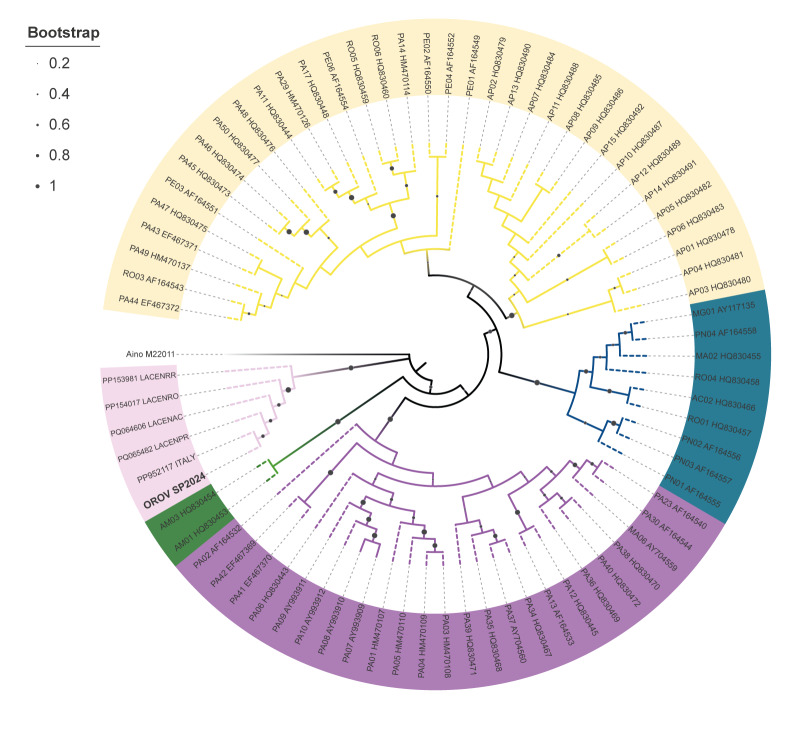
Phylogenetic analysis of Oropouche virus sequences from a traveller returning from Cuba, Spain, 2024 and Oropouche virus sequences from GenBank (n = 90)

Sequencing of the complete genome of OROV SP2024 (GenBank ID: PQ329253, PQ329254, PQ329255) presented a coverage of 99–100% (> 10 ×), generated with 7,951, 15,543 and 14,475 reads, respectively. Comparison of available N gene sequences from GenBank showed that OROV SP2024 was closely related to a new reassortant OROV strain from Brazil 2022–2024 and another one sequenced from an imported case of OROV in Italy 2024, which originated from Cuba, with a bootstrap support value of 1,000 (Figure 3).

OROV: Oropouche virus.

Neighbour-Joining phylogenetic tree based on the complete nucleotide sequences of the nucleoprotein gene of 91 OROV strains. Branches are colour-coded according to the genotype: genotype I (purple), genotype II (yellow), genotype III (blue), genotype IV (green) and the Brazil 2023–2024 group (pink). Aino virus represents the outgroup for the analysis.

## Outbreak response measures

Given the current low risk of local transmission, the clinicians were encouraged to focus on surveillance of travellers returning from endemic areas, with special attention to pregnant women. In Spain, molecular and serological diagnostic methods to carry out the detection of OROV suspected cases were implemented in the National Reference Laboratory. To support this strategy, an approach for evaluation of travellers with suspected OROV infection was developed and is presented in Supplement.

## Discussion

In the early summer of 2024, 13 imported cases of Oropouche fever were diagnosed in Spain. Cases among travellers returning from Latin American countries were also reported in Italy [13] and Germany, with 19 imported cases within the European Union (EU) [33]. The detection aligned with the epidemiological alert issued by PAHO, following a rise in OROV cases and its emergence in previously unaffected areas.

All confirmed cases in our study were travellers returning from Cuba, where a large OROV outbreak occurred in 2024 [11]. Clinically, patients presented mainly with fever, headache, myalgia, arthralgia, and asthenia, common symptoms in infections caused by other arboviruses, such as DENV, ZIKV and CHIKV [28,34]. This clinical similarity underscores the need for a precise differential diagnosis to avoid misidentification, particularly in non-endemic countries such as those in Europe, where these diseases are uncommon. None of the patients developed severe complications. This clinical course was consistent with previous reports describing OROV infection as having a generally favourable prognosis and rare fatal outcomes. Nevertheless, OROV possesses neuroinvasive potential, cases of aseptic meningitis and meningoencephalitis have been documented in previous outbreaks.

Three cases in our case series presented with a biphasic course of the illness, which can complicate clinical management by creating a false sense of recovery delaying the identification of potential complications [20]. Notably, two of these three patients developed neurological symptoms during the second phase of the illness. Recent studies indicate that the proportion of neurological complications in current OROV outbreaks in Latin America may be higher than previously described, including the association of Oropouche fever with Guillain-Barré syndrome [4,35]. Additionally, fatal cases were first documented during the 2024 epidemic in Brazil, even in previously healthy young patients [36]. Investigations into pregnancy-related adverse outcomes such as microcephaly and fetal demise due to maternal-fetal transmission are also ongoing [37-39]. These rare but severe sequelae underline the need for vigilance concerning neurological and obstetric complications [40].

Diagnosis relies often on RT-qPCR in serum or plasma collected during acute illness. However, duration of the viraemia can limit detection. In our report, analysis of urine samples enabled detection of viral RNA in three cases up to 2–3 weeks post-symptom onset (two with 22 dps and one with 24 dps) when blood and serum tests were negative. Although a small number of samples were included in our study, these results could indicate that in OROV infection, as in infections of other arboviruses, such as WNV, if the virus is excreted in urine, the viral load is higher and tends to persist for a longer period [41]. Testing other sample types than plasma and whole blood could be beneficial, when the clinical presentation is atypical and the time interval between exposure and sampling time is long. The virus may persist in urine and semen [42,43]. Most samples collected < 10 dps onset tested positive with RT-qPCR, indicating blood and serum could be reliable for early molecular diagnosis. Therefore, combining serum, whole blood and urine samples for RT-PCR analysis maximises diagnostic sensitivity. Additionally, in one patient negative by RT-qPCR, confirmation with neutralisation assay was successful, emphasising its value for diagnostic with convalescent samples, epidemiological investigations and seroprevalence studies.

The detection of imported OROV cases in Spain coincides with the emergence of other arboviruses, like WNV and DENV, in Europe, due to several factors like global travel, climate change, urbanisation and competent vector spread. The geographic expansion of OROV in 2022–2024 likely reflects these dynamics. The hypothesis of viral evolution is also gaining support: recent genomic analyses have identified the emergence of a previously undescribed reassortant variant of OROV that predominated during the large outbreak in the Amazon region in 2023–2024 [11]. Our genomic and phylogenetic analysis revealed high genetic identity between the isolate from Spain and OROV reassortant strains circulating in current Cuban and Brazilian outbreaks, and from imported cases in Italy [13,44].

In Europe, the current risk of local OROV transmission is low [45], because the main vector, *C. paraensis*, is absent in the continent [33]. Although European mosquito species have not been proven to be competent vectors, some *Culex* and *Aedes* species have shown the capacity to harbour and potentially transmit OROV under experimental conditions [46-48]. Further vector competence studies using the multiple viral strains are crucial. To date, no secondary cases or local OROV outbreaks have been documented in Europe, despite repeated importation of infected travellers.

Therefore, active surveillance, particularly among international travellers, and vector competence studies using the current OROV reassortant variant in European vectors are essential. Additionally, strengthening diagnostic capacity in European laboratories and raising awareness among healthcare professionals about this emerging disease are priorities.

Our study has certain limitations. It is based on a small number of cases (n = 13) identified during a short time frame and specific context, which limits the generalisability of the findings and only one isolate was sequenced. The patients were young–middle-aged, healthy travellers, and the observed clinical spectrum may not fully reflect disease manifestations in more vulnerable populations, such as individuals with comorbidities, other ages or pregnant people. Furthermore, not all specimen types were available at all stages of infection; in some cases, only late-phase samples were collected, limiting the ability to characterise viral load kinetics.

## Conclusion

Our findings highlight the diagnostic value of using various sample types and the importance of including OROV in differential diagnoses for febrile travellers from endemic areas. Given the potential for underdiagnosis and ongoing geographic expansion, heightened clinical awareness and enhanced surveillance in travellers are necessary.

## Data Availability

Generated sequences are available in GenBank (GenBank ID: PQ329253, PQ329254, PQ329255).

## Supplementary Data

546000 Supplementary Material
