# Supplementary material for: Case series of Oropouche fever among travellers returning from Cuba to Spain, 2024
Source: Euro Surveill. 2025 Oct 16;30(41):2400719. doi: 10.2807/1560-7917.ES.2025.30.41.2400719 (PMC12534779; doi:10.2807/1560-7917.ES.2025.30.41.2400719)
Supplement: Supplementary Material [file 24-00719_VAZQUEZ_Supplement.pdf]

Supplementary material

This supplementary material is hosted by Eurosurveillance as supporting information alongside the article *"Case series of Oropouche fever among travellers returning from Cuba to Spain, 2024"*, on behalf of the authors, who remain responsible for the accuracy and appropriateness of the content. The same standards for ethics, copyright, attributions and permissions as for the article apply. Supplements are not edited by Eurosurveillance and the journal is not responsible for the maintenance of any links or email addresses provided therein.

Content:

Annex: Proposed approach for the evaluation of travelers with suspected OROV infection

Annex: Proposed approach for the evaluation of travelers with suspected OROV infection

Aspect	Description
**Objective**	Approach for evaluating travelers with suspected Oropouche fever.
**1. Clinical History**	- **Travel History**: Investigate recent travel to endemic areas (mainly Cuba, Brazil, Peru), updated lists of affected territories should be consulted on a regular basis.<br>- **Clinical Symptoms**:<br>- Fever<br>- Headache<br>- Myalgia<br>- Arthralgia<br>- Nausea and vomiting<br>- Diarrhea<br>- Skin rash<br>- Retroocular pain<br>- Neurologic and hemorrhagic complications
**2. Criteria for suspected cases**	- Evaluation of symptoms in the context of mosquito exposure in endemic areas.<br>- Consider Oropouche fever if symptoms arise after traveling to areas with known virus circulation.
**3. Differential Diagnosis**	Warning signs and possible differentiating features:<br>- **Dengue**: hemorrhagic symptoms, severe muscle pain, evidence of plasma leakage (shock, respiratory distress).<br>- **Zika**: conjunctivitis, mild fever, and rash, especially during pregnancy.<br>- **Chikungunya**: severe joint pain that can last for weeks-months.<br>- **Oropouche Fever**: typical symptoms overlap with other arboviruses, OROV may have a biphasic illness
**4. Diagnosis**	- **Recommended Diagnostic Methods**:<br>- **RT-qPCR**: detection of viral RNA in blood, serum, LCR or preferably urine (shown to be a useful sample in travelers).<br>- **Serology**: neutralization tests in acute and convalescent sera.
**5. Management and Follow-Up**	- **Isolation and Notification**: patient isolation is not necessary. Notification of public health authorities if infection is informed.<br>- **Consider pregnancy status** in women of gestational age<br>- **Surveillance**: clinical monitoring to assess disease progression and detect late complications such as neurologic and hemorrhagic symptoms.
**6. Prevention and Education**	- **Inform** travelers about risks and preventive measures (use of repellents, mosquito nets).<br>- **Encourage** seeking medical attention if symptoms arise after returning from endemic areas.
